# Complications following miniplate insertion in maxillofacial fractures: a systematic review

**DOI:** 10.12688/f1000research.159017.2

**Published:** 2025-02-25

**Authors:** Bramasto Purbo Sejati, Ahmad Kusumaatmaja, Maria Goreti Widiastuti, Tetiana Haniastuti

**Affiliations:** 1 Departemnt of Oral and maxillofacial Surgery, Universitas Gadjah Mada, Yogyakarta, Special Region of Yogyakarta, 55284, Indonesia; 2 Departement of Physics, Universitas Gadjah Mada, Yogyakarta, Special Region of Yogyakarta, 55284, Indonesia; 3 Departement of Oral Bilogy, Universitas Gadjah Mada, Yogyakarta, Special Region of Yogyakarta, 55284, Indonesia

**Keywords:** systematic review; miniplate; complications; maxillofacial fractures.

## Abstract

**Background:**

Maxillofacial fractures, frequently arising from road traffic incidents, falls, and acts of interpersonal aggression, are a considerable public health issue, exhibiting diverse epidemiological patterns according to demographic factors. The application of miniplates for fracture stabilization is a recognized technique, with innovative methods such as 3D plate systems emerging. Nonetheless, consequences including infections and hardware malfunctions persist. This systematic review seeks to present current evidence regarding the complications linked to miniplate placement in maxillofacial fractures over the last ten years.

**Methods:**

A systematic review was performed in accordance with PRISMA principles. Databases such as the Cochrane Library, PubMed, and Scopus were examined from September 2014 to September 2024. Studies documenting problems related to miniplate placement were included, without language constraints. The ROBINS-I tool was utilized for non-randomized studies, whereas the Cochrane risk of bias tool was applied to randomized controlled trials.

**Results:**

From 2,289 initially found studies, 56 satisfied the inclusion criteria. Among these, 28 employed interventional designs, whilst the remaining 28 were observational research. The predominant problems documented in several investigations encompassed infection, wound dehiscence, malocclusion, paraesthesia, malunion/non-union, segment movement, hardware failure, and palpable hardware. Advanced methodologies such as 3D plate systems and locking mechanisms were linked to diminished complication rates.

**Conclusion:**

This systematic analysis presents a decade of updated research about problems associated with miniplate placement in maxillofacial fractures. Novel methodologies such as 3D plate systems and locking mechanisms demonstrate promise in mitigating problems relative to conventional techniques. These findings can facilitate informed decision-making in clinical practice. Additional study utilizing standardized outcomes and prospective designs is essential to enhance comprehension of the long-term effects of miniplate utilization.

## Introduction

Maxillofacial fractures represent a considerable public health issue, frequently arising from road traffic accidents (RTAs), falls, and interpersonal violence, with RTAs being the primary cause in many countries, including Jordan and India. ^1, 2^ The epidemiology of these fractures differs by demographics, exhibiting a greater occurrence in males, especially within the 21-30 age range. ^2^ In Germany, there is a discernible increase in the frequency of maxillofacial trauma procedures, underscoring the necessity for efficient surgical interventions. ^3^ In low- and middle-income countries, enhancing surgeon training is essential for improving care and outcomes in cranio-maxillofacial injuries. ^4^ A multidisciplinary approach is crucial for the proper management of these complicated injuries.

Miniplate insertion is widely used in surgery, particularly in orthodontics and fracture stabilization. The insertion procedure generally requires 25 to 30 minutes, with research indicating an overall success rate of 95.5% for miniplates utilized in orthodontic treatments. ^5^ The stability of these miniplates is greatly affected by the quality and amount of cortical bone at the insertion site, rendering them advantageous in regions with restricted bone availability. ^6^ Three-dimensional imaging has improved miniplate placement accuracy, leading to better surgical outcomes and reduced operation time. ^6^ Miniplate fixation is a dependable technique for establishing solid skeletal anchoring and promoting healing. ^5, 7^

The available literature reveals a research gap concerning problems associated with miniplate implantation in craniofacial and orthognathic operations, indicating a necessity for more comprehensive studies. Systematic reviews highlight complications such as infections, plate exposure, and removal preference, with reported complication rates varying up to 32.5%. ^8^ The average removal time is variable, spanning from 5.5 to 10.7 months, which suggests the absence of defined monitoring techniques. ^9^ Most studies are retrospective, limiting conclusions on long-term outcomes and risk factors of miniplate use. ^9^ This highlights the need for future research with more substantial, prospective cohorts and standardized outcome measures to further understanding of the implications of miniplate insertion and related problems in clinical practice. ^10^

This systematic review updates evidence on complications of miniplate insertion in maxillofacial fractures. Additionally, the results of this study could benefit healthcare practitioners and patients in making an informed decision regarding the applicability and potential complications.

## Methods

### Data Sources and Searches

This systematic review was performed based on PRISMA guidelines on systemic reviews and meta-analyzes (PROSPERO: CRD42024612052). ^11^ The Cochrane Library, PubMed, and Scopus were examined from September 1, 2014, to September 1, 2024. We implement a decade-long trend to guarantee the innovative technique. We utilize the keyword combinations as follows: miniplate AND complications AND maxillofacial. Table 1 delineated a combination of various search techniques. Furthermore, relevant papers that satisfied the inclusion criteria were manually identified within each retrieved study.

**Table 1.** Search strategy.

Database	Keywords
PubMed	(“miniplate”[All Fields] OR “miniplates”[All Fields] OR “miniplating”[All Fields]) AND (“complicances”[All Fields] OR “complicate”[All Fields] OR “complicated”[All Fields] OR “complicates”[All Fields] OR “complicating”[All Fields] OR “complication”[All Fields] OR “complication s”[All Fields] OR “complications”[MeSH Subheading] OR “complications”[All Fields]) AND “maxillofacial”[All Fields]
Scopus	(ALL (miniplate) AND ALL (complications) AND ALL (maxillofacial))
Cochrane Library	miniplate AND complications AND maxillofacial

### Study Selection

We included a comprehensive original study detailing the complications associated with miniplate placement in maxillofacial fractures. No linguistic constraints were imposed. Studies were considered irrespective of the languages utilized, provided that English translations were accessible. Two reviewers (B.P.S. and A.K.) conducted separate evaluations of the titles and abstracts of possibly qualifying articles. All differences were deliberated with the third investigator (T.H).

### Data Extraction

Two independent assessors (B.P.S. and A.K.) extracted data and resolved discrepancies. We incorporated the subsequent data: 1) Attributes of the included studies (e.g., first author’s name and publication year); 2) demographic attributes of the patient population (e.g., age, male percentage, and participant count in each group); 3) intervention attributes (e.g., type); and 4) outcomes. Disputes were settled by dialogue with the corresponding author (T.H.) until a consensus was achieved.

### Descriptions of outcome Measures

The reported outcome was complications following miniplate insertion in maxillofacial fractures. We endeavored to reach the original authors to acquire further or missing information through email.

### Risk of Bias Assessment

The quality of the included studies was evaluated according to the criteria outlined in the Cochrane Handbook for Systematic Reviews of Interventions. ^12^ The Cochrane risk of bias assessment was utilized for the randomized control trials. ^13^ We evaluated the risk of bias in non-randomized studies utilizing the checklist for prevalence studies. ^14^ All studies were evaluated by two independent reviewers (B.P.S. and A.K.). All disputes have been resolved during the consensus meeting.

### Data Synthesis and Analysis

This systematic review emphasizes the synthesis and analysis of data to summarize and interpret findings from individual studies. The review initially delineates attributes, including design, sample size, interventions, and outcomes. The synthesis entails a critical evaluation of the research’ quality, addressing methodological rigor and identifying any biases or limitations.

## Results

### Search Result

**Figure 1.**
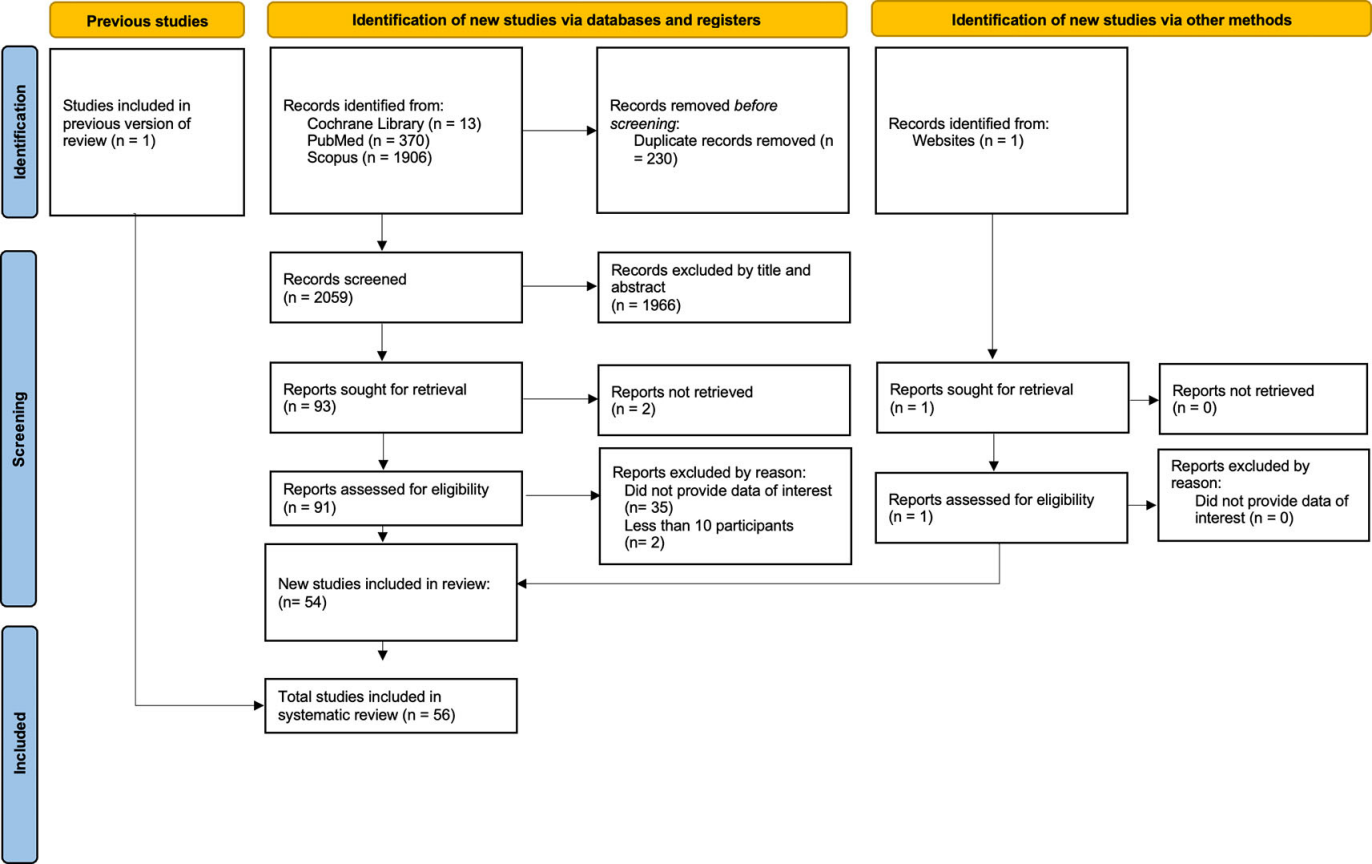
PRISMA flow diagram.

Figure 1 illustrates the electronic search procedure. We initially detected 2,289 articles. Of these, 2,196 duplicate papers or irrelevant studies were eliminated. Ninety-one papers were identified for additional investigation. Thirty-seven research were omitted from this list due to insufficient relevant data (Appendix Table 1). Two additional papers were acquired: one from a prior literature review and the other from a website search. We incorporated a total of 56 studies that fulfilled the inclusion criteria. ^15– 70^

### Study Characteristics

The attributes of the studies included have been captured in Table 2. Out of 56 investigations, 28 utilized an interventional design, including randomized controlled trials (RCTs), whereas the other 28 were observational studies, comprising cohort designs. India was the most prevalent country of study origin. This distribution represents a balance between experimental and observational research methodologies within the dataset, offering a comprehensive foundation for analyzing the investigated phenomena.

The majority of research contrasted conventional miniplate types with innovative methods, including 3D plate systems. Additionally, several studies investigated the effectiveness of locking compared to non-locking strategies in similar interventions. The most commonly reported complications included infection, wound dehiscence, malocclusion, paraesthesia, malunion/non-union, segment movement, hardware failure, and palpable hardware. These results underscore the variety of potential complications linked to the use of miniplates in clinical practice. Other details of results is provided in Table 2 extended data.

### Risk of Bias Assessment of Included Studies

Result of risk of bias assessment were described in Table 3 for RCTs studies. Twelve and ten studies addressed concerns about sufficient randomization and incomplete outcome data. five studies used adequate concealment of allocation. Participants and personnel in four studies were blind to treatment assignment, while assessors were unaware in four studies.

**Table 3.** Risk of bias RoB 1.0.

No.	First Authors, year	Random sequence generation	Allocation concealment	Blinding of participants and personnel	Blinding of outcome assessment	Incomplete outcome data addressed
1	Adhikari, 2021	L	U	U	U	L
2	Agarwal, 2014	U	U	U	U	U
3	Agnihotri, 2014	L	U	U	U	U
4	Al-Moraissi, 2015	L	L	L	L	L
5	Camino Junior, 2017	L	U	U	U	L
6	Kanubaddy, 2016	L	U	U	U	L
7	Kumar, 2023	L	L	L	L	L
8	Mannan, 2018	L	L	L	L	U
9	Mathew, 2022	L	U	U	U	L
10	Rai, 2018	L	U	U	U	L
11	Sehgal, 2014	L	L	L	L	L
12	Tiwari, 2019	L	U	U	U	L
13	Yang, 2015	L	L	U	U	L

L = Low risk; H = High risk; U = Unclear risk of bias.

The quality assessment of risk of bias for non-randomized studies can be seen in Table 4. overall studies yielded low risk of bias.

**Table 4.** Risk of bias checklist for prevalence studies.

No.	First Authors, year	1	2	3	4	5	6	7	8	9
1	Aggarwal, 2017	Y	Y	Y	Y	Y	Y	Y	Y	Y
2	Amjad, 2020	Y	Y	Y	Y	Y	Y	Y	Y	Y
3	Bhagat, 2021	Y	Y	Y	Y	Y	Y	Y	Y	Y
4	Bhatt, 2015	Y	Y	Y	Y	Y	Y	Y	Y	Y
5	Bohner, 2020	Y	Y	Y	Y	Y	Y	Y	Y	Y
6	Burkhard, 2020	Y	Y	Y	Y	Y	Y	Y	Y	Y
7	Carricondo, 2018	Y	Y	Y	Y	Y	Y	Y	Y	Y
8	Daif, 2014	Y	Y	Y	Y	Y	Y	Y	Y	Y
9	Dediol, 2014	Y	Y	Y	Y	Y	Y	Y	Y	Y
10	Fani, 2020	Y	Y	Y	Y	Y	Y	Y	Y	Y
11	Ferrari, 2018	Y	Y	Y	Y	Y	Y	Y	Y	Y
12	Fernandes, 2022	Y	Y	Y	Y	Y	Y	Y	Y	Y
13	Gamit, 2024	Y	Y	Y	Y	Y	Y	Y	Y	Y
14	Ghezta, 2016	Y	Y	Y	Y	Y	Y	Y	Y	Y
15	Graillon, 2021	Y	Y	Y	Y	Y	Y	Y	Y	Y
16	Kaushik, 2020	Y	Y	Y	Y	Y	Y	Y	Y	Y
17	Kerdoud, 2021	Y	Y	Y	Y	Y	Y	Y	Y	Y
18	Khan, 2020	Y	Y	Y	Y	Y	Y	Y	Y	Y
19	Khandelwal, 2019	Y	Y	Y	Y	Y	Y	Y	Y	Y
20	Kreutzer. 2023	Y	Y	Y	Y	Y	Y	Y	Y	Y
21	Mishra, 2019	Y	Y	Y	Y	Y	Y	Y	Y	Y
22	Mondal, 2019	Y	Y	Y	Y	Y	Y	Y	Y	Y
23	Palani, 2021	Y	Y	Y	Y	Y	Y	Y	Y	Y
24	Pfister, 2024	Y	Y	Y	Y	Y	Y	Y	Y	Y
25	Rahpeyma, 2014	Y	Y	Y	Y	Y	Y	Y	Y	Y
26	Rai, 2021	Y	Y	Y	Y	Y	Y	Y	Y	Y
27	Ribeiro-Junior, 2018	Y	Y	Y	Y	Y	Y	Y	Y	Y
28	Rohit, 2019	Y	Y	Y	Y	Y	Y	Y	Y	Y
29	Saha, 2015	Y	Y	Y	Y	Y	Y	Y	Y	Y
30	Sakong, 2021	Y	Y	Y	Y	Y	Y	Y	Y	Y
31	Sarepally, 2022	Y	Y	Y	Y	Y	Y	Y	Y	Y
32	Shaik, 2014	Y	Y	Y	Y	Y	Y	Y	Y	Y
33	Sikora, 2020	Y	Y	Y	Y	Y	Y	Y	Y	Y
34	Singh, 2016	Y	Y	Y	Y	Y	Y	Y	Y	Y
35	Singh, 2020a	Y	Y	Y	Y	Y	Y	Y	Y	Y
36	Singh, 2020b	Y	Y	Y	Y	Y	Y	Y	Y	Y
37	Spinelli, 2016	Y	Y	Y	Y	Y	Y	Y	Y	Y
38	Sukegawa, 2019a	Y	Y	Y	Y	Y	Y	Y	Y	Y
39	Sukegawa, 2019b	Y	Y	Y	Y	Y	Y	Y	Y	Y
40	Sukegawa, 2020	Y	Y	Y	Y	Y	Y	Y	Y	Y
41	Sweta, 2022	Y	Y	Y	Y	Y	Y	Y	Y	Y
42	Vashistha, 2017	Y	Y	Y	Y	Y	Y	Y	Y	Y
43	Yadav, 2020	Y	Y	Y	Y	Y	Y	Y	Y	Y

Y = yes; N = no; U = unclear; NA = not applicable.

## Discussion

This systematic review presents updated ten-year evidence about complications associated with miniplate insertion in maxillofacial fractures. The results of our study may assist healthcare practitioners and patients in making informed decisions about the applicability and potential difficulties associated with miniplate deployment. This study employed a rigorous methodology, ensuring high-confidence findings.

Infection is a notable problem subsequent to the placement of miniplates in maxillofacial surgery. Consistent with prior research, the overall infection rate linked to miniplates is roughly 13.3%, which is the primary reason for their removal, representing about 2.9% of cases in one meta-analysis. ^9, 71^ A study on orthodontic anchorage miniplates revealed that 17.3% of the placed miniplates developed infections, influenced significantly by the proximity to the mucogingival junction and the frequency of dental hygiene. ^72^ In addition, one study reported the microbiological research indicated that Staphylococcus aureus was the primary pathogen at infected locations, underscoring the necessity for regular microbial evaluations to inform therapy. ^73^ These findings highlight the necessity of monitoring and mitigating infection risks associated with miniplate utilization.

The utilization of 3D miniplates in managing mandibular fractures has been associated to a reduced incidence of complications relative to traditional systems. Studies demonstrate that 3D miniplates enhance stability and diminish operational duration, resulting in superior intraoperative results and markedly fewer problems, including enhanced biting force and fracture stability. ^74^ A retrospective investigation of 336 patients indicated that merely 8.03% encountered minor problems, whereas significant difficulties arose in only 1.49% of instances. ^75^ A study on patient-specific 3D-printed miniplates exhibited exceptional precision in fixing and effective osseous union, devoid of material fractures or plate exposure. ^76^ The adaptability and effectiveness of 3D miniplates enhance their successful application in the treatment of mandibular fractures, underscoring their advantages compared to conventional techniques. ^77^

Several limitations must be acknowledged. First, a meta-analysis was not performed owing to the heterogeneity of the included studies. Secondly, the patient demographics and treatment durations differed among research, highlighting the necessity for meticulous evaluation when selecting protocols to pay attention to. Third, we include less prospective study. The evaluation encompasses a decade-long range to concentrate on the most recent methodologies in this field of research.

## Conclusion and implications

This study’s findings indicate that the majority of research has concentrated on contrasting conventional miniplate types with more advanced ways, such as 3D plate systems, while also assessing the efficacy of locking versus non-locking procedures. The extensive range of documented complications—such as infection, wound dehiscence, malocclusion, paraesthesia, malunion or non-union, segment movement, hardware failure, and palpable hardware—illustrates the clinical difficulties associated with miniplate implantation. Careful selection of miniplate technology and surgical techniques is crucial for minimizing complications and improving outcomes.

## Author contributions

Conceived and designed the experiments: BPS TH. Analyzed the data: BPS AK. Wrote the paper: BPS TH. Designed search strategies: BPS AK TH. Critically reviewed the manuscript for important intellectual content: BPS AK MGW TH. Read and approved the final version: BPS AK MGW TH. Guarantors: BPS TH.

## Ethics and consent

Ethical approval and consent were not required.

## Data Availability

Underlying data- No data are associated with this article. Extended Data Zenodo: Supplementary data, 10.5281/zenodo.14064447. ^78^ This project contains the following underlying data:
1. Reporting guidelines, PRISMA checklist 2. Appendix Table 1. List of the excluded studies Data are available under the terms of the Creative Commons Zero “No rights reserved” data waiver (Creative Commons Attribution 4.0 International). Zenodo: Characteristics of included studies, 10.5281/zenodo.14207706. ^79^ This project contains the following underlying data:
•
Table 2 Characteristics of included studies Data are available under the terms of the Creative Commons Zero “No rights reserved” data waiver (Creative Commons Attribution 4.0 International).
