# Supplementary material for: Complications following miniplate insertion in maxillofacial fractures: a systematic review
Source: F1000Res. 2025 Mar 6;13:1507. Originally published 2024 Dec 9. [Version 3] doi: 10.12688/f1000research.159017.3 (PMC11869201; doi:10.12688/f1000research.159017.3)
Supplement: Peer review history

# Reviewer report: Reviewer response for version 2

**DOI:** 10.5256/f1000research.178440.r368385

**Published:** 2025-03-03

**Authors:** Mohammad Adhitya Latief

**Affiliations:** 1 University of Indonesia, Depok, West Java, Indonesia

I appreciate the time and effort you have taken to address my comments and improve the quality of the manuscript. After careful evaluation, I am pleased to inform you that your manuscript has fulfill has fulfilled my request for correction as per my expectation. The revisions have satisfactorily addressed the concerns raised during the review process that I suggested, and no further modifications are required at this stage.

Are the rationale for, and objectives of, the Systematic Review clearly stated?

Partly

Is the statistical analysis and its interpretation appropriate?

No

If this is a Living Systematic Review, is the ‘living’ method appropriate and is the search schedule clearly defined and justified? (‘Living Systematic Review’ or a variation of this term should be included in the title.)

Not applicable

Are sufficient details of the methods and analysis provided to allow replication by others?

No

Are the conclusions drawn adequately supported by the results presented in the review?

Partly

Reviewer Expertise:

trauma and fractures management in oral maxillofacial

I confirm that I have read this submission and believe that I have an appropriate level of expertise to confirm that it is of an acceptable scientific standard.

# Reviewer report: Reviewer response for version 2

**DOI:** 10.5256/f1000research.178440.r368384

**Published:** 2025-02-26

**Authors:** Mohamed Jaber

**Affiliations:** 1 Ajman University, Ajman, United Arab Emirates

I reviewed the modified version of the manuscript, the authors incorporated all my suggestions for improvements, thus I have no further comments to make.

 Final recommendation: Approve.

Are the rationale for, and objectives of, the Systematic Review clearly stated?

Yes

Is the statistical analysis and its interpretation appropriate?

Partly

If this is a Living Systematic Review, is the ‘living’ method appropriate and is the search schedule clearly defined and justified? (‘Living Systematic Review’ or a variation of this term should be included in the title.)

Not applicable

Are sufficient details of the methods and analysis provided to allow replication by others?

Partly

Are the conclusions drawn adequately supported by the results presented in the review?

Partly

Reviewer Expertise:

Maxillofacial injuries, Oral cancer and precancer, dental education

I confirm that I have read this submission and believe that I have an appropriate level of expertise to confirm that it is of an acceptable scientific standard.

# Reviewer report: Reviewer response for version 1

**DOI:** 10.5256/f1000research.174686.r348231

**Published:** 2025-02-14

**Authors:** Indra Mulyawan

**Affiliations:** 1 Oral and Maxillofacial Surgery, Airlangga University, Surabaya, East Java, Indonesia

This article is quite good and complete, has met the requirements of a good article, only needs to be sharpened in the background regarding the intent and direction of the purpose of this research. only a few articles reviewed are confirmed to be in accordance with the theme of the article. 

 Just need to provides more references to have a strength reason why we have to use the miniplate, how the best way to use the miniplates and also how to take care the complications. It is very useful for an oral maxillofacial surgeon to decide the best therapy in maxillofacial fractures.

Are the rationale for, and objectives of, the Systematic Review clearly stated?

Yes

Is the statistical analysis and its interpretation appropriate?

Yes

If this is a Living Systematic Review, is the ‘living’ method appropriate and is the search schedule clearly defined and justified? (‘Living Systematic Review’ or a variation of this term should be included in the title.)

Yes

Are sufficient details of the methods and analysis provided to allow replication by others?

Yes

Are the conclusions drawn adequately supported by the results presented in the review?

Yes

Reviewer Expertise:

oral and maxillofacial surgery

I confirm that I have read this submission and believe that I have an appropriate level of expertise to confirm that it is of an acceptable scientific standard.

# Response

**Published:** 2025-02-18

**Authors:** bramasto purbo sejati

**Affiliations:** OMFS, Universitas Gadjah Mada, Yogyakarta, Special Region of Yogyakarta, Indonesia

Response: Thank you for review and acknowledgement.

# Reviewer report: Reviewer response for version 1

**DOI:** 10.5256/f1000research.174686.r348230

**Published:** 2025-01-22

**Authors:** Mohammad Adhitya Latief

**Affiliations:** 1 University of Indonesia, Depok, West Java, Indonesia

Power:

1. Comprehensive Review: The paper systematically reviews complications associated with miniplate insertion in maxillofacial fractures over a decade, providing a broad and updated perspective, as mention on methods that data examined from September 1, 2014, to September 1, 2024. ​
2. Diverse Data Sources: It utilizes multiple databases (Cochrane Library, PubMed, Scopus) and includes studies without language constraints, ensuring a wide range of data as mentioned in methods section, data source and searches. ​
3. Methodological Rigor: The review follows PRISMA guidelines and employs robust tools (ROBINS-I, Cochrane risk of bias tool) for assessing study quality, enhancing the reliability of findings. ​
4. Balanced Study Designs: The inclusion of both interventional and observational studies offers a comprehensive analysis of the phenomena. This distribution represents a balance between experimental and observational research methodologies within the dataset, offering a comprehensive foundation for analyzing the investigated phenomena.​
5. Focus on Novel Techniques: The paper highlights advanced methodologies like 3D plate systems and locking mechanisms, which show promise in reducing complications. ​

Weakness:

1. Heterogeneity: The included studies exhibit significant variability in patient demographics, treatment durations, and methodologies, which limits the ability to perform a meta-analysis. ​One of the example is decision to include the use of miniplate in orthognatic surgery, Because the title is focussing in complication of miniplate in maxillofacial fractures, but the samples used in this research also include miniplate in orthognatic surgery which is very different in outcomes or surgery result.
2. Lack of Standardization: The absence of standardized outcome measures across studies makes it challenging to compare results directly. Although I cant find table 2 as result in the article, I could tell that it is challenging since there will be differences outcome because the different usage in fracture cases and orthognatic cases
3. Limited Prospective Data: There is a need for more prospective studies to better understand the long-term effects of miniplate utilization. ​ As mention in article that Additional study utilizing standardized outcomes and prospective designs is essential to enhance comprehension of the long-term effects of miniplate utilization
4. Potential Bias: Despite using robust tools, the risk of bias in some studies remains unclear, which could affect the overall conclusions. We might correct this by using statistic to eliminate any factor that has potential bias, but unfortunately I cant find any of them in this article.

Overall, the paper provides valuable insights but also highlights the need for more standardized and prospective research to fully understand the implications of miniplate insertion in maxillofacial fractures. However,  In my opinion the article provides a comprehensive exploration of the role and applications of miniplates in craniofacial surgery and Oral and maxillofacial field procedures. It delves into their potential complications, offering insights into their effectiveness in stabilizing fractures, facilitating orthodontic surgery, and supporting reconstructive surgeries. This article broadens our understanding of miniplate application, guiding clinicians in selecting appropriate systems and optimizing outcomes. By addressing both benefits and limitations, it serves as a valuable resource for advancing knowledge in the field and improving patient care.

Are the rationale for, and objectives of, the Systematic Review clearly stated?

Partly

Is the statistical analysis and its interpretation appropriate?

No

If this is a Living Systematic Review, is the ‘living’ method appropriate and is the search schedule clearly defined and justified? (‘Living Systematic Review’ or a variation of this term should be included in the title.)

Not applicable

Are sufficient details of the methods and analysis provided to allow replication by others?

No

Are the conclusions drawn adequately supported by the results presented in the review?

Partly

Reviewer Expertise:

trauma and fractures management in oral maxillofacial

I confirm that I have read this submission and believe that I have an appropriate level of expertise to confirm that it is of an acceptable scientific standard, however I have significant reservations, as outlined above.

# Response

**Published:** 2025-02-18

**Authors:** bramasto purbo sejati

**Affiliations:** OMFS, Universitas Gadjah Mada, Yogyakarta, Special Region of Yogyakarta, Indonesia

Reviewer 2:

 Power:

 Comprehensive Review: The paper systematically reviews complications associated with miniplate insertion in maxillofacial fractures over a decade, providing a broad and updated perspective, as mention on methods that data examined from September 1, 2014, to September 1, 2024. ​

 Diverse Data Sources: It utilizes multiple databases (Cochrane Library, PubMed, Scopus) and includes studies without language constraints, ensuring a wide range of data as mentioned in methods section, data source and searches. ​

 Methodological Rigor: The review follows PRISMA guidelines and employs robust tools (ROBINS-I, Cochrane risk of bias tool) for assessing study quality, enhancing the reliability of findings. ​

 Balanced Study Designs: The inclusion of both interventional and observational studies offers a comprehensive analysis of the phenomena. This distribution represents a balance between experimental and observational research methodologies within the dataset, offering a comprehensive foundation for analyzing the investigated phenomena.​

 Focus on Novel Techniques: The paper highlights advanced methodologies like 3D plate systems and locking mechanisms, which show promise in reducing complications. ​

**Response**: Thank you for your review and acknowledgment.

 Weakness:

 Heterogeneity: The included studies exhibit significant variability in patient demographics, treatment durations, and methodologies, which limits the ability to perform a meta-analysis. ​One of the example is decision to include the use of miniplate in orthognatic surgery, Because the title is focussing in complication of miniplate in maxillofacial fractures, but the samples used in this research also include miniplate in orthognatic surgery which is very different in outcomes or surgery result.

**Response**: Thank you for review. We acknowledge this in our limitation of study.

 Lack of Standardization: The absence of standardized outcome measures across studies makes it challenging to compare results directly. Although I cant find table 2 as result in the article, I could tell that it is challenging since there will be differences outcome because the different usage in fracture cases and orthognatic cases

**Response**: We agree with your suggestion. Due to the significant heterogeneity of the included studies, we were unable to conduct a meta-analysis; therefore, we can only perform a systematic review.

 Limited Prospective Data: There is a need for more prospective studies to better understand the long-term effects of miniplate utilization. ​ As mention in article that Additional study utilizing standardized outcomes and prospective designs is essential to enhance comprehension of the long-term effects of miniplate utilization

**Response**: We acknowledge this as the limitation of the study.

 Potential Bias: Despite using robust tools, the risk of bias in some studies remains unclear, which could affect the overall conclusions. We might correct this by using statistic to eliminate any factor that has potential bias, but unfortunately I cant find any of them in this article.

**Response**: We utilize the risk of bias assessment tool based on the Joanna Briggs Institute assessment scale. According to the grading scale, we cannot draw an overall conclusion; instead, we will categorize each item with responses of “yes,” “no,” and “unclear.”

 Overall, the paper provides valuable insights but also highlights the need for more standardized and prospective research to fully understand the implications of miniplate insertion in maxillofacial fractures. However,  In my opinion the article provides a comprehensive exploration of the role and applications of miniplates in craniofacial surgery and Oral and maxillofacial field procedures. It delves into their potential complications, offering insights into their effectiveness in stabilizing fractures, facilitating orthodontic surgery, and supporting reconstructive surgeries. This article broadens our understanding of miniplate application, guiding clinicians in selecting appropriate systems and optimizing outcomes. By addressing both benefits and limitations, it serves as a valuable resource for advancing knowledge in the field and improving patient care.

**Response**: We sincerely appreciate the reviewer’s favorable remarks and perceptive recommendations. In accordance with the given comments, we have meticulously revised the manuscript to improve clarity and coherence.

# Reviewer report: Reviewer response for version 1

**DOI:** 10.5256/f1000research.174686.r355750

**Published:** 2025-01-16

**Authors:** Mohamed Jaber

**Affiliations:** 1 Ajman University, Ajman, United Arab Emirates

**Review Report for Manuscript Submitted to F1000Research**

**Title**: Complications following miniplate insertion in maxillofacial fractures: A systematic review.

 The manuscript is relevant to the field of maxillofacial surgery and explores an important area: complications associated with miniplate insertion. It aligns with the interests of F1000Research, particularly in presenting evidence-based insights useful for clinical practice. However, significant improvements are required for it to meet the journal's standards of rigor and clarity.

**Key findings**

- The manuscript reviews ten years of data on complications following miniplate insertion, providing valuable insights into trends and advanced methodologies.
- The focus on novel techniques such as 3D plate systems highlights advancements with potential to reduce complications.
- The authors adhere to PRISMA guidelines and employ robust tools like the ROBINS-I and Cochrane risk of bias tools to evaluate study quality.
- The discussion emphasizes the need for standardized outcomes and prospective studies, which is vital for advancing clinical practices.

**Limitations and Suggestions for Improvement:**

- The results and discussion sections could benefit from deeper statistical and comparative analyses. For example: provide quantitative comparisons between traditional and advanced methodologies. Detail the statistical significance of the findings related to complication rates.
- The search strategy appears robust, but details on the inclusion/exclusion criteria for studies are insufficient. A clearer explanation of why certain studies were excluded (e.g., irrelevance or quality issues) would enhance transparency.
- Some sections, particularly the introduction and discussion, are repetitive. Consolidating overlapping points and presenting a more concise narrative will improve readability.
- The inclusion of figures or summary tables (e.g., comparison of complication rates by technique) would better communicate the findings.
- While the PRISMA framework was followed, the authors did not conduct a meta-analysis. If heterogeneity precluded this, it should be explicitly stated and justified in the text.
- The language is generally clear but needs refinement to eliminate redundancy and improve flow. For example, the repeated mention of infection rates and their significance across sections could be consolidated.
- Certain sentences are overly technical, which might hinder accessibility for a broader audience. Simplifying terminology without losing accuracy is recommended.
- The manuscript lacks a detailed comparison of its findings with those of previous reviews or meta-analyses. Highlighting how this review advances knowledge or contradicts earlier work would enhance its contribution.
- A more robust discussion of study limitations is necessary, particularly concerning variability in patient demographics and differences in surgical expertise, healthcare infrastructure across studies, and retrospective study designs in many included papers.

**Key Recommendations:**

1. Include statistical analyses or at least a detailed qualitative comparison of complication rates.
2. Streamline the text, especially in sections with overlapping content.
3. Add summary tables or figures to improve data visualization.
4. Clarify methodological choices, particularly regarding the heterogeneity of included studies.
5. Expand on the implications of the findings, both clinical and research-oriented.

**Suitability for indexing:**

 The manuscript has potential but requires revisions to strengthen its methodological rigor, depth of analysis, and presentation. With these changes, it could serve as a valuable resource for clinicians and researchers in maxillofacial surgery.

 I recommend **major revisions** prior to indexing.

 Thank you

Are the rationale for, and objectives of, the Systematic Review clearly stated?

Yes

Is the statistical analysis and its interpretation appropriate?

Partly

If this is a Living Systematic Review, is the ‘living’ method appropriate and is the search schedule clearly defined and justified? (‘Living Systematic Review’ or a variation of this term should be included in the title.)

Not applicable

Are sufficient details of the methods and analysis provided to allow replication by others?

Partly

Are the conclusions drawn adequately supported by the results presented in the review?

Partly

Reviewer Expertise:

Maxillofacial injuries, Oral cancer and precancer, dental education

I confirm that I have read this submission and believe that I have an appropriate level of expertise to state that I do not consider it to be of an acceptable scientific standard, for reasons outlined above.

# Response

**Published:** 2025-02-18

**Authors:** bramasto purbo sejati

**Affiliations:** OMFS, Universitas Gadjah Mada, Yogyakarta, Special Region of Yogyakarta, Indonesia

Reviewer 1:

 Review Report for Manuscript Submitted to F1000Research

 Title: Complications following miniplate insertion in maxillofacial fractures: A systematic review.

 The manuscript is relevant to the field of maxillofacial surgery and explores an important area: complications associated with miniplate insertion. It aligns with the interests of F1000Research, particularly in presenting evidence-based insights useful for clinical practice. However, significant improvements are required for it to meet the journal's standards of rigor and clarity.

 Key findings

 The manuscript reviews ten years of data on complications following miniplate insertion, providing valuable insights into trends and advanced methodologies.

 The focus on novel techniques such as 3D plate systems highlights advancements with potential to reduce complications.

 The authors adhere to PRISMA guidelines and employ robust tools like the ROBINS-I and Cochrane risk of bias tools to evaluate study quality.

 The discussion emphasizes the need for standardized outcomes and prospective studies, which is vital for advancing clinical practices.

**Response**: Thank you for your insightful assessment and constructive recommendations. We value your acknowledgment of the manuscript's significance to maxillofacial surgery and its prospective impact on clinical practice.

 ----------

 Limitations and Suggestions for Improvement:

 The results and discussion sections could benefit from deeper statistical and comparative analyses. For example: provide quantitative comparisons between traditional and advanced methodologies. Detail the statistical significance of the findings related to complication rates.

**Response**: Thank you for your insightful feedback. We recognize the significance of comprehensive statistical and comparative analysis. Nonetheless, the heterogeneity of the included studies—characterized by differences in research design, patient demographics, intervention procedures, and outcome measures—precluded the possibility of conducting a meta-analysis or offering direct statistical comparisons between traditional and advanced approaches. We performed a systematic review to qualitatively synthesize the existing evidence. This limitation has been explicitly addressed in the manuscript's Limitation section to maintain transparency. We have meticulously analyzed the findings to offer significant insights into the trends and possible ramifications of the examined approaches.

 ----------

 The search strategy appears robust, but details on the inclusion/exclusion criteria for studies are insufficient. A clearer explanation of why certain studies were excluded (e.g., irrelevance or quality issues) would enhance transparency.

**Response**: Thank you for your input. We value your inquiry regarding the criteria for inclusion and exclusion. We wish to highlight that the justifications for study exclusion have already been detailed in Appendix Table 1.

 ----------

 Some sections, particularly the introduction and discussion, are repetitive. Consolidating overlapping points and presenting a more concise narrative will improve readability.

**Response**: We have carefully reviewed the manuscript and guaranteed that the updated version is more succinct, with superfluous points eliminated, especially in the Introduction and Discussion sections. The revised version now offers a more coherent and concise narrative while preserving the requisite analytical depth. We value your recommendation and assert that these modifications improve the manuscript's general readability.

 ----------

 The inclusion of figures or summary tables (e.g., comparison of complication rates by technique) would better communicate the findings.

**Response**: To improve clarity and effectively convey our data, we have incorporated Table 2, which presents a comparative summary of complication rates by technique. Nonetheless, owing to the heterogeneity of the data—characterized by discrepancies in study designs, outcome definitions, and patient populations—we were unable to produce a forest plot or do a meta-analysis. The manuscript acknowledges this limitation.

 ----------

 While the PRISMA framework was followed, the authors did not conduct a meta-analysis. If heterogeneity precluded this, it should be explicitly stated and justified in the text.

**Response**: The manuscript acknowledges this limitation.

 ----------

 The language is generally clear but needs refinement to eliminate redundancy and improve flow. For example, the repeated mention of infection rates and their significance across sections could be consolidated.

**Response**: The document has been carefully reviewed and the text has been updated to remove redundancy and enhance overall coherence. We have synthesized redundant references to infection rates and their significance to provide a more concise and consistent presentation.

 ----------

 Certain sentences are overly technical, which might hinder accessibility for a broader audience. Simplifying terminology without losing accuracy is recommended.

**Response**: We have carefully reviewed the manuscript and refined excessively technical sentences to improve readability while preserving scientific precision. We have refined terminology when necessary to maintain accessibility for a wider audience while preserving the integrity of our analysis.

 ----------

 The manuscript lacks a detailed comparison of its findings with those of previous reviews or meta-analyses. Highlighting how this review advances knowledge or contradicts earlier work would enhance its contribution.

**Response**: To mitigate this risk, we have included citations of prior systematic reviews in the Introduction to contextualize our analysis. Furthermore, we have ensured that our discourse emphasizes how our findings correlate with and enhance the current body of research.

 ----------

 A more robust discussion of study limitations is necessary, particularly concerning variability in patient demographics and differences in surgical expertise, healthcare infrastructure across studies, and retrospective study designs in many included papers.

**Response**: We have enhanced the Limitations section to offer a more comprehensive analysis of the probable sources of variability in our selected studies. We now examine variations in patient demographics, surgical proficiency, and healthcare infrastructure that may affect the reported outcomes. Furthermore, we recognize the influence of retrospective study designs in numerous included articles and its consequences for data dependability and bias.

 ----------

 Key Recommendations:

 Include statistical analyses or at least a detailed qualitative comparison of complication rates.

 Streamline the text, especially in sections with overlapping content.

 Add summary tables or figures to improve data visualization.

 Clarify methodological choices, particularly regarding the heterogeneity of included studies.

 Expand on the implications of the findings, both clinical and research-oriented.

**Response**: I appreciate your valuable suggestions. We have carefully evaluated each point and updated the manuscript accordingly. To fulfill the requirement for statistical or qualitative comparisons, we have presented a comprehensive qualitative comparison of complication rates in Table 2, as a meta-analysis was impracticable due to data heterogeneity. We have refined the language, especially in the Introduction and Discussion, to remove redundancy and enhance clarity. Table 2 has been incorporated to summarize significant findings for improved data presentation; nevertheless, a forest plot could not be produced due to the diversity of the included research. Furthermore, we have elucidated our methodological selections, especially about the influence of sample heterogeneity, in the Methods and Limitations sections. We have broadened the discourse on the clinical and research ramifications of our findings to emphasize their importance and prospective research avenues. We value your astute recommendations, which have enhanced the manuscript.

 ----------

 ​Suitability for indexing:

 The manuscript has potential but requires revisions to strengthen its methodological rigor, depth of analysis, and presentation. With these changes, it could serve as a valuable resource for clinicians and researchers in maxillofacial surgery.

 I recommend major revisions prior to indexing.

**Response**: Thank you for your assessment and insightful feedback. We have carefully revised the manuscript to enhance its methodological rigor, analytical depth, and presentation, in accordance with your recommendations. The modifications have been emphasized in red for your convenience. We anticipate that the improvements align with your expectations and augment the manuscript's utility as a significant resource for physicians and researchers in maxillofacial surgery.
